# Inheritance of Resistance to Sorghum Shoot Fly, *Atherigona soccata* in Sorghum, *Sorghum bicolor* (L.) Moench

**DOI:** 10.3389/fpls.2016.00543

**Published:** 2016-04-27

**Authors:** Riyazaddin Mohammed, Ashok Kumar Are, Rajendra Sudhakar Munghate, Ramaiah Bhavanasi, Kavi Kishor B. Polavarapu, Hari Chand Sharma

**Affiliations:** ^1^International Crops Research Institute for the Semi-Arid Tropics (ICRISAT)Patancheru, India; ^2^Department of Genetics, Osmania UniversityHyderabad, India

**Keywords:** sorghum, *Atherigona soccata*, resistance, combining ability, heritability, general combining ability, specific combining ability

## Abstract

Sorghum production is affected by a wide array of biotic constraints, of which sorghum shoot fly, *Atherigona soccata* is the most important pest, which severely damages the sorghum crop during the seedling stage. Host plant resistance is one of the major components to control sorghum shoot fly, *A. soccata*. To understand the nature of gene action for inheritance of shoot fly resistance, we evaluated 10 parents, 45 F_1_'s and their reciprocals in replicated trials during the rainy and postrainy seasons. The genotypes ICSV 700, Phule Anuradha, ICSV 25019, PS 35805, IS 2123, IS 2146, and IS 18551 exhibited resistance to shoot fly damage across seasons. Crosses between susceptible parents were preferred for egg laying by the shoot fly females, resulting in a susceptible reaction. ICSV 700, ICSV 25019, PS 35805, IS 2123, IS 2146, and IS 18551 exhibited significant and negative general combining ability (*gca*) effects for oviposition, deadheart incidence, and overall resistance score. The plant morphological traits associated with expression of resistance/susceptibility to shoot fly damage such as leaf glossiness, plant vigor, and leafsheath pigmentation also showed significant *gca* effects by these genotypes, suggesting the potential for use as a selection criterion to breed for resistance to shoot fly, *A. soccata*. ICSV 700, Phule Anuradha, IS 2146 and IS 18551 with significant positive *gca* effects for trichome density can also be utilized in improving sorghums for shoot fly resistance. The parents involved in hybrids with negative specific combining ability (*sca*) effects for shoot fly resistance traits can be used in developing sorghum hybrids with adaptation to postrainy season. The significant reciprocal effects of combining abilities for oviposition, leaf glossy score and trichome density suggested the influence of cytoplasmic factors in inheritance of shoot fly resistance. Higher values of variance due to specific combining ability (σ^2^s), dominance variance (σ^2^d), and lower predictability ratios than the variance due to general combining ability (σ^2^g) and additive variance (σ^2^a) for shoot fly resistance traits indicated the predominance of dominance type of gene action, whereas trichome density, leaf glossy score, and plant vigor score with high σ^2^g, additive variance, predictability ratio, and the ratio of general combining ability to the specific combining ability showed predominance of additive type of gene action indicating importance of heterosis breeding followed by simple selection in breeding shoot fly-resistant sorghums. Most of the traits exhibited high broadsense heritability, indicating high inheritance of shoot fly resistance traits.

## Introduction

Sorghum [*Sorghum bicolor* (L.) Moench] is an important food and fodder crop of dryland agriculture. It has a wide range of adaptability to various agro-ecological conditions in the semi-arid tropics (SAT). It is a staple food crop for millions of poor people living in the SAT regions of Asia and Africa (Ashok Kumar et al., 2011). It is cultivated on marginal, fragile, and drought-prone environments in the semi-arid tropics. Sorghum is also grown for forage, and is fed to animals as a green chop, silage or hay. Sorghum grain is one of the major ingredients in poultry and cattle feed in USA, China and Australia (Bramel-Cox et al., 1995). It is also emerging as an important bio-fuel crop. India is the largest sorghum grower in the world with an average productivity of 854.4 kg ha^−1^ (FAO, 2014). In the dry land agriculture, the yield and quality of sorghum produced is affected by a wide array of biotic and abiotic constraints. Several insect pests damage sorghum from seedling stage to maturity. Around 150 insect pests attack sorghum (Jotwani et al., 1980; Sharma, 1993), of which sorghum shoot fly, *Atherigona soccata* is a serious pest that reduces sorghum production in the semi-arid tropics. Due to shoot fly damage, a loss of 80–90% of grain, and 68% of fodder yield was recorded in India (Balikai and Bhagwat, 2009; Kahate et al., 2014). Identifying sorghum genotypes with stable shoot fly resistance is highly important as it will help to reduce the cost of cultivation and stabilize the yields.

Shoot fly, *A. soccata* attacks sorghum at 7-30 days after seedling emergence (DAE). It lays white elongated cigar shaped eggs on the lower surface of the leaf, parallel to leaf midrib (Padmaja et al., 2010). Maggot emerges from the egg in 2 days, reaches the central whorl, cuts the central leaf, and starts feeding on the decaying leaf tissue of central whorl. As a result, the central whorl dries off resulting in a typical deadheart (Deeming, 1971; Dhillon et al., 2006a). It completes its life cycle in 17–21 days (Sharma et al., 2003). Many approaches have been used to minimize the losses caused by shoot fly, including agronomic practices, natural enemies, synthetic insecticides, and host plant resistance (Sharma, 1993; Kumar et al., 2008), but implementation of all these practices is not always feasible.

Host plant resistance (HPR) is one of the most economic and practical means for controlling shoot fly damage because it does not involve any extra cost to the farmers or require application skills in pest control techniques (Sharma, 1985; Dhillon et al., 2006a). It is also compatible with other methods of pest control. The negative effects of resistant genotypes on insect populations are continuous and cumulative over time. Reduction in pest abundance through HPR can also enhance the effectiveness of natural enemies, and reduces the need to apply pesticides (Sharma, 1993).

In view of serious economic losses due to shoot fly damage, improvement of sorghum for shoot fly resistance is one of the major goals in sorghum improvement programs. Although several improved varieties and hybrids have been developed and released, the yield gains at farmers' level are minimal (Sharma et al., 2003). Therefore, there is a need to combine shoot fly resistance with high grain yield to increase the productivity of this crop. Because of the poor understanding of inheritance of shoot fly resistance, sorghum improvement for resistance to this pest have not been very effective (Doggett et al., 1970; Riyazaddin et al., 2015a). Combining ability studies is needed to identify better combiners and develop superior hybrids that would be helpful in understanding the nature of gene action and inheritance of quantitative traits (Singh and Chaudhary, 1985; Goyal and Kumar, 1991; Mohammed et al., 2015).

Diallel crosses have been widely used in genetic research to investigate the inheritance of important agronomical and morphological traits. Studies on combining ability estimates are useful to understand the nature of genetic variance, and to predict the relative performance of different lines in hybrid combinations. Information on the nature and magnitude of gene action is important in understanding the genetic potential of a population, and deciding the breeding procedure to be adopted in a given population (Prabhakar and Raut, 2010). Several researchers worked on developing sorghum genotypes with resistance to shoot fly, but the genetic gains were quite low (Ashok Kumar et al., 2011). Therefore, the present studies were carried out to understand the nature of gene action of shoot fly resistance and morphological traits, and their inheritance to study the general and specific combining abilities of parents and crosses, respectively, to establish appropriate strategies for developing sorghum genotypes with improved resistance to shoot fly, *A. soccata*.

## Materials and methods

### Experimental material

The experiments were conducted at the International Crops research Institute for the Semi-Arid Tropics (ICRISAT), Patancheru-502 324, Telangana, India. Based on performance of the genotypes in the field (Riyazaddin et al., 2015a,b), 10 sorghum genotypes exhibiting high levels of resistance (ICSV 25019, PS 35805, IS 2123, IS 2146, and IS 18551) or susceptibility (CSV 15 and Swarna) to shoot fly; and ICSV 700, M 35-1, and Phule Anuradha with moderate levels of shoot fly resistance (Annexure I in Supplementary Material) which are genetically diverse were selected for the crossing program. These genotypes were used for crossing in a full diallel fashion i.e., crossing in all possible combinations including reciprocals, to test the hybrid vigor of crosses and combining abilities, and the reciprocal effects of parental genotypes. Crossing of 10 selected parents in a full diallel fashion generated 45 direct crosses, and 45 reciprocal crosses (90 F_1_'s).

The test material consisting of 10 parents and 90 F_1_'s was sown in three replications in a randomized complete block design (RCBD). One set of replicated trial was raised under protected conditions during the 2013 rainy and postrainy seasons. The test genotypes were sown in the field in 2.0 m row length, row to row spacing of 75 cm and with a spacing of 10 cm in-between the plants within a row. A basal dose of ammonium phosphate @100 kg/ha was applied to the field. Parents were sown in two rows each, and the F_1_'s in a single row. The test material was tested under high shoot fly pressure using interlard fishmeal technique (Soto, 1974; Sharma et al., 1992). Thinning was carried out at 7 days after seedling emergence. Earthing up and topdressing with urea (@100 kg/ha) were carried out at 30 DAE. Hand weeding was carried out whenever necessary. Furrow irrigation was given to the experimental material during the 2013 postrainy season.

## Observations

### Shoot fly damage parameters

Data were recorded on plants with shoot fly eggs and number of shoot fly eggs per plant at 14 DAE, and shoot fly deadhearts at 21 DAE, and expressed as the percentage of plants with shoot fly eggs, number of shoot fly eggs per plant, and percentage of plants with deadhearts. Overall resistance score was recorded on a 1-9 scale before harvesting (1 = plants with < 10% deadhearts and uniform tillers and harvestable panicles, and 9 = plants with >80% deadhearts, and a few or no productive tillers) (Sharma et al., 1992).

### Morphological characteristics

Leaf glossiness was evaluated visually on a 1–5 scale (1 = highly glossy, and 5 = non-glossy) at 10–12 DAE (fifth leaf stage), when the expression of this trait is most apparent in the early morning hours (Sharma and Nwanze, 1997). Leafsheath pigmentation was visually scored on a 1-3 rating scale (1 = leafsheath with deep pink pigmentation, and 3 = green leafsheath and no pink pigmentation) at 7 DAE (Dhillon et al., 2006b). Seedling vigor was recorded at 10 DAE on 1–3 scale (1 = high seedling vigor, and 3 = poor seedling vigor) (Sharma et al., 1992). Waxy bloom was visually scored on 1–3 scale (1 = slightly waxy, and 3 = completely waxy) at the flag leaf stage of the crop. Plant color was evaluated visually on a 1–2 scale (1 = non-tan; red colored plant, and 2 = tan; colorless plant).

The density of trichomes on both the surfaces of leaf was recorded at 12 DAE by taking a 2.5 cm^2^ portion from the center of the fifth leaf (Maiti and Bidinger, 1979). The leaf samples were taken from three plants at random, de-stained overnight with 2:1 of acetic acid: alcohol. The de-stained leaf is stored in 90% lactic acid, and observed at 10× magnification under a stereomicroscope. Trichomes on the leaf surface were counted and expressed as numbers of trichomes in a 10× microscopic field (density per microscopic field).

### Statistical analysis

The data were subjected to analysis of variance (ANOVA) using GenStat® 13th version (GenStat, 2010). Significance of the differences between the genotypes was judged by *F*-test, while the genotypic means were compared by least significant difference (LSD) at *P* ≤ 0.05. Diallel analysis of parents and F_1_'s was carried out according to the Griffing's method 1 and model 1 (Griffing, 1956), which partitions the total variation into the general combining ability (*gca*) effects that provide the genetic nature of the parents, and the specific combining ability (*sca*) effects that provide information about the performance of hybrids, using Windowstat software (Indostat Services, 2004). The coefficient of variation at phenotypic and genotypic levels was estimated using the formula adopted by Johnson et al. (1955) and predictability ratio using Baker (1978).

## Results

### Expression of resistance to shoot fly, *A. soccata* by the F_1_ hybrids (10 × 10 diallel) in comparison with the parents

The variance ratio for 10 parents, 45 F_1_'s along with 45 reciprocal crosses for all the traits studied were significant at *P* ≤ 0.01, with few exceptions. Variance ratio of plants with shoot fly eggs (%), and number of shoot fly eggs/plant were significant only in the postrainy season (Table 1).

**Table 1:** **Expression of resistance to sorghum shoot fly, ***A. soccata*** in sorghum (ICRISAT, Patancheru, 2013–14)**.

**Pedigree**	**Plants with shoot fly eggs (%)**		**Number of shoot fly eggs/plant**		**Shoot fly deadhearts (%)**		**ORS**	
	**2013 R**	**2013 PR**	**2013 R**	**2013 PR**	**2013 R**	**2013 PR**	**2013 R**	**2013 PR**
**PARENTS**								
ICSV 700	92.24	16.55	2.33	1.13	66.44	7.48	6.33	6.67
Phule Anuradha	96.29	10.02	2.15	1.00	75.92	8.06	5.00	6.33
M 35-1	91.41	18.25	1.70	1.06	83.72	17.77	8.33	4.00
CSV 15	100.00	44.49	3.57	1.22	83.33	40.40	8.00	6.33
ICSV 25019	69.73	16.53	1.31	1.05	21.45	6.39	5.00	4.33
PS 35805	96.19	10.21	1.81	1.06	44.62	2.17	4.33	4.00
IS 2123	81.33	10.44	2.27	1.00	60.07	4.30	6.00	4.00
IS 2146	94.80	17.72	2.24	1.13	54.59	5.32	6.00	5.33
IS 18551	89.78	6.30	2.12	1.33	60.21	3.96	6.00	4.00
Swarna	93.60	84.57	1.72	1.38	76.45	53.05	8.67	8.33
**DIRECT CROSSES**								
ICSV 700 X Phule Anuradha	100.00	9.39	1.71	0.67	88.64	6.67	5.00	7.00
ICSV 700 X M 35-1	100.00	36.57	1.94	1.00	89.56	32.87	5.67	6.00
ICSV 700 X CSV 15	93.94	30.81	1.92	1.08	80.68	26.64	7.00	7.67
ICSV 700 X ICSV 25019	86.90	22.62	2.17	0.89	73.81	7.54	5.67	5.33
ICSV 700 X PS 35805	80.91	27.78	2.15	1.33	70.91	37.04	4.67	5.67
ICSV 700 X IS 2123	87.88	35.91	3.09	1.00	85.61	15.28	6.00	6.33
ICSV 700 X IS 2146	93.94	33.43	1.65	1.39	74.68	10.17	6.67	5.00
ICSV 700 X IS 18551	100.00	35.81	2.10	1.00	83.33	17.22	6.00	7.00
ICSV 700 X Swarna	93.33	56.78	2.10	1.29	93.33	34.30	7.67	7.00
Phule Anuradha X M 35-1	90.48	3.03	1.56	0.33	90.48	3.03	6.67	6.33
Phule Anuradha X CSV 15	88.89	53.70	2.17	1.00	92.59	35.19	6.00	6.00
Phule Anuradha X ICSV 25019	100.00	47.13	1.26	1.00	96.97	25.05	6.00	6.00
Phule Anuradha X PS 35805	100.00	30.71	1.42	1.00	94.44	19.20	6.33	5.00
Phule Anuradha X IS 2123	100.00	28.69	2.15	1.17	85.86	20.36	5.67	4.67
Phule Anuradha X IS 2146	88.33	54.98	1.93	1.00	92.46	41.29	5.33	6.00
Phule Anuradha X IS 18551	100.00	15.58	2.24	1.00	77.78	14.29	5.67	6.33
Phule Anuradha X Swarna	100.00	65.02	2.21	1.47	94.44	51.86	7.00	6.00
M 35-1 X CSV 15	100.00	48.68	2.30	1.17	91.67	52.38	6.67	5.67
M 35-1 X ICSV 25019	91.67	4.17	2.24	0.33	91.38	12.50	4.67	6.00
M 35-1 X PS 35805	100.00	10.37	1.87	0.67	76.67	6.67	6.33	4.00
M 35-1 X IS 2123	86.77	14.54	2.15	1.00	66.27	17.50	6.00	5.67
M 35-1 X IS 2146	95.83	24.44	2.29	0.83	82.37	24.44	6.00	5.67
M 35-1 X IS 18551	96.08	30.00	3.26	1.83	72.16	28.89	6.00	5.00
M 35-1 X Swarna	91.53	29.44	2.04	1.33	72.75	6.67	5.33	6.67
CSV 15 X ICSV 25019	94.44	70.91	1.80	2.03	94.44	55.45	7.00	6.67
CSV 15 X PS 35805	100.00	83.82	1.54	1.07	93.89	37.88	6.00	7.33
CSV 15 X IS 2123	100.00	21.09	1.76	1.42	85.19	14.42	5.00	5.00
CSV 15 X IS 2146	100.00	72.89	2.16	1.29	89.56	63.36	6.00	5.67
CSV 15 X IS 18551	96.30	60.15	2.14	1.23	97.44	42.12	6.00	7.00
CSV 15 X Swarna	93.33	58.18	1.71	1.56	86.67	68.48	7.67	7.00
ICSV 25019 X PS 35805	85.19	16.98	1.84	1.00	44.44	8.89	5.67	6.33
ICSV 25019 X IS 2123	90.48	2.08	2.47	0.33	61.69	20.83	5.00	5.67
ICSV 25019 X IS 2146	100.00	21.59	2.56	1.00	83.33	11.36	4.67	4.33
ICSV 25019 X IS 18551	95.83	7.54	2.19	0.67	95.83	20.71	5.00	5.00
ICSV 25019 X Swarna	86.77	53.52	1.89	1.27	79.37	46.48	7.33	7.67
PS 35805 X IS 2123	90.48	15.76	1.83	0.67	69.05	12.73	4.33	5.67
PS 35805 X IS 2146	90.11	18.52	2.08	0.83	68.42	14.81	5.00	7.00
PS 35805 X IS 18551	87.83	8.10	2.49	0.67	67.20	5.90	4.33	5.67
PS 35805 X Swarna	97.44	49.66	2.06	1.28	66.80	28.28	6.67	7.00
IS 2123 X IS 2146	66.67	12.63	1.33	1.00	66.67	7.87	6.33	6.67
IS 2123 X IS 18551	91.67	15.74	2.18	0.67	77.22	11.57	5.67	6.00
IS 2123 X Swarna	100.00	51.67	1.85	1.41	92.59	24.17	4.33	5.67
IS 2146 X IS 18551	100.00	6.49	2.09	0.67	87.88	2.56	6.33	4.67
IS 2146 X Swarna	93.64	66.32	1.89	1.22	87.58	51.91	5.67	5.33
IS 18551 X Swarna	74.81	61.34	3.44	1.32	85.93	46.41	6.00	5.33
**RECIPROCAL CROSSES**								
Phule Anuradha X ICSV 700	100.00	26.85	2.21	1.17	78.33	9.72	5.00	5.33
M 35-1 X ICSV 700	100.00	23.65	1.42	1.08	89.26	12.22	5.33	7.00
M 35-1 X Phule Anuradha	100.00	11.11	1.92	0.44	76.07	0.00	6.33	5.67
CSV 15 X ICSV 700	100.00	91.67	2.27	1.19	91.90	45.83	5.33	6.00
CSV 15 X Phule Anuradha	100.00	37.04	1.39	0.88	93.33	39.15	6.00	6.33
CSV 15 X M 35-1	96.97	55.39	2.05	1.15	96.97	61.45	5.67	6.00
ICSV 25019 X ICSV 700	93.33	13.69	2.33	0.67	76.67	8.93	5.00	6.33
ICSV 25019 X Phule Anuradha	84.85	20.00	1.56	1.11	49.90	13.33	5.67	6.00
ICSV 25019 X M 35-1	100.00	27.36	2.14	1.07	91.53	9.26	5.67	5.33
ICSV 25019 X CSV 15	100.00	99.29	2.97	1.56	85.79	46.19	6.33	7.67
PS 35805 X ICSV 700	100.00	16.30	1.88	1.17	56.88	5.56	5.33	6.00
PS 35805 X Phule Anuradha	83.33	4.17	3.08	0.33	53.17	0.00	5.67	6.00
PS 35805 X M 35-1	100.00	33.86	2.23	1.00	89.74	17.99	6.00	6.00
PS 35805 X CSV 15	88.89	20.63	2.14	0.83	77.78	30.16	7.00	7.67
PS 35805 X ICSV 25019	100.00	25.71	1.89	1.00	75.79	0.00	6.33	6.00
IS 2123 X ICSV 700	96.97	23.93	1.82	1.17	63.54	12.26	6.67	6.33
IS 2123 X Phule Anuradha	100.00	28.10	2.29	1.33	80.16	28.69	7.00	4.67
IS 2123 X M 35-1	97.62	42.06	2.33	1.00	84.92	12.96	5.33	6.33
IS 2123 X CSV 15	100.00	62.64	2.80	1.14	95.24	47.19	6.67	6.00
IS 2123 X ICSV 25019	95.83	25.16	2.19	1.00	64.96	16.23	3.67	5.33
IS 2123 X PS 35805	100.00	15.15	1.67	0.67	82.15	15.15	5.00	6.00
IS 2146 X ICSV 700	100.00	16.50	3.47	1.00	93.33	0.00	6.33	5.00
IS 2146 X Phule Anuradha	93.94	12.96	1.87	0.67	90.91	6.48	5.67	5.67
IS 2146 X M 35-1	87.04	16.62	1.25	1.00	57.83	18.22	6.33	5.67
IS 2146 X CSV 15	100.00	59.43	2.27	1.13	84.26	49.20	5.00	4.67
IS 2146 X ICSV 25019	100.00	13.22	1.38	1.17	86.61	12.96	3.50	5.00
IS 2146 X PS 35805	96.97	24.36	2.29	1.00	90.24	11.79	5.33	4.67
IS 2146 X IS 2123	100.00	9.71	2.27	1.33	73.61	16.85	6.00	4.33
IS 18551 X ICSV 700	100.00	24.16	1.64	1.25	69.44	10.94	6.67	6.67
IS 18551 X Phule Anuradha	90.28	15.00	1.73	0.67	60.00	6.67	6.33	5.33
IS 18551 X M 35-1	75.76	30.28	2.00	1.33	63.64	3.33	6.67	6.67
IS 18551 X CSV 15	100.00	33.27	1.72	1.23	91.67	26.88	6.67	4.67
IS 18551 X ICSV 25019	91.67	9.09	1.59	0.44	83.06	24.58	5.00	5.33
IS 18551 X PS 35805	100.00	25.57	2.35	1.07	78.70	13.26	5.00	5.67
IS 18551 X IS 2123	82.22	10.36	2.22	1.17	72.22	5.59	6.00	4.67
IS 18551 X IS 2146	96.97	23.61	2.19	1.53	74.46	13.06	5.33	6.00
Swarna X ICSV 700	100.00	89.17	2.23	1.31	98.04	58.33	4.67	6.33
Swarna X Phule Anuradha	100.00	60.71	1.78	1.12	90.00	44.05	6.33	6.00
Swarna X M 35-1	100.00	65.29	1.49	0.94	96.97	32.42	6.00	6.00
Swarna X CSV 15	100.00	81.96	1.95	1.26	100.00	65.75	8.00	6.67
Swarna X ICSV 25019	100.00	57.41	2.01	1.54	96.97	52.78	7.33	7.33
Swarna X PS 35805	100.00	63.64	2.25	1.29	91.56	25.76	6.00	7.33
Swarna X IS 2123	100.00	63.26	2.37	1.63	74.44	37.88	7.00	6.00
Swarna X IS 2146	100.00	43.59	2.17	1.29	100.00	48.29	6.33	6.33
Swarna X IS 18551	100.00	67.33	2.43	1.47	100.00	47.48	6.00	6.33
Mean	94.59	33.80	2.08	1.08	79.99	23.57	5.92	5.88
SE ±	6.35	10.76	0.43	0.26	10.09	10.38	0.54	0.61
Vr (99, 198)	1.25^*NS*^	4.79^**^	1.14^*NS*^	1.45^**^	2.06^**^	3.05^**^	3.06^**^	2.31^**^
LSD (P 0.05)	17.72	30.00	1.19	0.72	28.14	28.93	1.51	1.71

** *F-test significant at P = 0.01; R, rainy season; PR, postrainy season; NS, non-significant F value; ORS, Overall resistance score: 1–9 ranking with 1 = plants with uniform tillers and harvestable panicles; 9 = plants with a few or no productive tillers*.

Very high levels of oviposition (2–4 eggs per plant) were recorded during the rainy season as compared to the postrainy season (1–2 eggs/plant) (Table 1). During the rainy season, the genotypes ICSV 25019 (69.73%) and IS 2123 (81.33%), the direct crosses IS 2123 X IS 2146 (66.67%), and IS 18551 X Swarna (74.81%), and the reciprocal cross IS 18551 X M 35-1 (75.76%) exhibited lower oviposition as compared to that of susceptible check Swarna with 93.60% of plants with shoot fly eggs. In the postrainy season, almost all the crosses exhibited lower percentage of plants with shoot fly eggs than the susceptible check Swarna, with few exceptions. Higher ovipostion was observed in the susceptible genotypes CSV 15 and Swarna and in crosses where these genotypes were involved as either of the parents, with 2-3 shoot fly eggs/plant across the seasons.

The percentage of plants with shoot fly deadhearts in the parents varied from 21.45–83.72% during rainy season, and 3.96–53.05% in the postrainy season (Table 1); and for crosses, the deadheart percentage ranged from 44.44 to 100% in the rainy season, and 0.00–68.48% during the postrainy season. The genotypes ICSV 700, Phule Anuradha, ICSV 25019, PS 35805, IS 2123, and IS 2146 exhibited lower shoot fly damage than CSV 15 and Swarna. M 35-1 showed a susceptible reaction in the rainy season and exhibited resistant nature in the postrainy season. Thirty-three crosses exhibited resistance to shoot fly across seasons, and PS 35805 and IS 18551 were involved as one of the parents in most of these crosses.

M 35-1 X Swarna, PS 35805 X Swarna, IS 18551 X ICSV 700, IS 18551 X Phule Anuradha, and ICSV 700 X IS 2146 exhibited antibiosis resistance mechanism i.e., lower numbers of plants with shoot fly deadhearts than the plants with shoot fly eggs (Table 1). Twenty-four crosses showed antibiosis as a mechanism of resistance to shoot fly in the rainy season. The direct crosses ICSV 700 X IS 2123, ICSV 700 X IS 18551, CSV 15 X PS 35805, CSV 15 X IS 18551, PS 35805 X Swarna, IS 2123 X Swarna, and reciprocal crosses CSV 15 X ICSV 700, ICSV 25019 X CSV 15, IS 2123 X M 35-1, IS 18551 X M 35-1, Swarna X M 35-1, Swarna X PS 35805, and Swarna X IS 2123 exhibited lower shoot fly deadhearts than the plants with shoot fly eggs, indicating antibiosis as mechanism of resistance to shoot fly during the postrainy season.

### Morphological traits

The parents ICSV 700, Phule Anuradha, IS 2123, IS 2146 and IS 18551, and the 29 crosses were glossy with high plant vigor, and had leafsheath pigmentation and high trichome density on the abaxial and adaxial leaf surfaces (Table 2). The hybrids ICSV 700 X M 35-1, ICSV 700 X PS 35805, ICSV 700 X IS 2146, Phule Anuradha X M 35-1, M 35-1 X Phule Anuradha, M 35-1 X IS 2146, M 35-1 X IS 18551, IS 2146 X Phule Anuradha, and IS 18551 X Swarna expressed the leaf glossiness, leafsheath pigmentation, high vigor and high trichome density only in the rainy season; whereas the crosses ICSV 700 X CSV 15, M 35-1 X PS 35805, PS 35805 X IS 2123, PS 35805 X IS 18551, IS 2123 X M 35-1, IS 2123 X IS 2146 expressed these traits only in the postrainy season.

**Table 2:** **Morphological characteristics of sorghum (parents and F_**1**_ crosses) evaluated for resistance to sorghum shoot fly, ***A. soccata*** (ICRISAT, Patancheru, 2013–14)**.

**Pedigree**	**Leaf glossy score**		**Plant vigor score**		**Leafsheath pigmentation**		**Trichome density on abaxial leaf surface**		**Trichome density on adaxial leaf surface**		**Waxy bloom**		**Plant color**	
	**2013 R**	**2013 PR**	**2013 R**	**2013 PR**	**2013 R**	**2013 PR**	**2013 R**	**2013 PR**	**2013 R**	**2013 PR**	**2013 R**	**2013 PR**	**2013 R**	**2013 PR**
**PARENTS**														
ICSV 700	2.67	1.00	1.33	1.00	1.67	1.00	139.00	89.56	162.67	109.22	1.33	2.00	2.00	2.00
Phule Anuradha	1.50	2.67	1.00	1.33	1.50	2.00	73.33	62.56	98.22	64.00	1.67	2.00	1.00	1.00
M 35-1	3.67	3.00	2.67	1.33	1.67	2.00	83.78	70.67	85.44	57.67	2.00	2.00	1.00	1.00
CSV 15	3.33	5.00	2.00	3.00	1.67	3.00	0.00	0.00	0.00	0.00	3.00	2.67	2.00	2.00
ICSV 25019	2.33	3.33	1.00	2.33	1.00	2.00	45.56	47.56	64.78	45.33	3.00	3.00	2.00	2.00
PS 35805	4.00	3.33	2.33	2.33	1.67	2.00	64.78	61.22	74.11	72.89	3.00	3.00	2.00	2.00
IS 2123	3.67	1.00	2.33	1.00	2.33	2.00	58.78	51.44	60.11	59.28	1.00	2.00	1.00	1.00
IS 2146	2.00	1.33	1.33	1.00	1.67	1.67	106.89	84.28	120.67	97.61	1.00	2.00	1.00	1.00
IS 18551	3.33	1.00	2.00	1.00	1.67	1.33	84.11	84.56	97.56	101.56	1.00	1.00	1.00	1.00
Swarna	3.67	5.00	2.00	2.33	2.00	2.00	33.89	7.11	32.22	13.33	3.00	3.00	1.00	1.00
**DIRECT CROSSES**														
ICSV 700 X Phule Anuradha	3.67	2.33	2.00	1.67	1.67	1.67	59.67	59.06	92.33	72.11	1.00	2.00	1.00	1.00
ICSV 700 X M 35-1	3.00	3.33	1.33	2.33	2.00	2.00	66.44	83.11	108.67	104.78	1.00	2.00	1.00	1.00
ICSV 700 X CSV 15	3.67	3.33	2.33	2.00	1.33	2.00	51.67	54.78	89.00	101.28	1.67	3.00	2.00	2.00
ICSV 700 X ICSV 25019	2.67	2.67	1.67	1.33	1.00	2.00	68.33	75.89	112.22	102.67	1.33	2.00	2.00	2.00
ICSV 700 X PS 35805	2.67	4.00	1.33	2.33	1.00	2.33	49.56	52.33	74.78	63.56	1.33	2.33	2.00	2.00
ICSV 700 X IS 2123	3.33	3.00	1.67	2.00	1.67	2.00	49.22	64.11	78.89	87.78	1.00	2.00	1.00	1.00
ICSV 700 X IS 2146	2.67	3.00	1.00	2.33	1.33	2.00	54.11	71.44	93.00	74.00	1.33	1.67	1.00	1.00
ICSV 700 X IS 18551	2.33	2.33	1.33	1.33	1.33	1.67	65.11	90.78	102.67	96.44	1.00	1.33	1.00	1.00
ICSV 700 X Swarna	3.00	4.67	1.50	2.33	2.00	2.00	67.11	58.11	104.78	74.78	1.67	2.33	1.00	1.00
Phule Anuradha X M 35-1	2.67	3.33	1.33	2.33	1.67	2.67	51.33	58.56	68.11	72.22	1.00	2.33	1.00	1.00
Phule Anuradha X CSV 15	4.00	4.00	2.00	1.67	1.67	2.67	34.22	36.50	48.00	38.94	1.67	2.67	1.00	1.00
Phule Anuradha X ICSV 25019	3.33	3.33	1.33	2.00	1.33	2.33	52.11	56.00	69.56	56.67	1.33	2.33	1.00	1.00
Phule Anuradha X PS 35805	3.00	3.67	1.67	2.00	2.00	2.33	56.00	53.11	68.22	64.56	1.33	2.00	1.00	1.00
Phule Anuradha X IS 2123	3.00	3.00	2.00	2.00	1.33	2.00	45.00	67.11	61.56	66.22	1.00	1.67	1.00	1.00
Phule Anuradha X IS 2146	3.33	3.67	2.00	1.67	1.67	2.33	81.22	69.56	98.22	88.22	1.67	1.67	1.00	1.00
Phule Anuradha X IS 18551	2.00	3.33	1.00	1.33	1.33	2.00	75.00	81.44	95.22	98.00	1.67	1.00	1.00	1.00
Phule Anuradha X Swarna	4.67	4.67	2.33	2.00	2.00	2.67	27.28	9.17	35.22	18.17	2.33	2.67	1.00	1.00
M 35-1 X CSV 15	3.67	4.33	2.00	2.67	1.33	2.33	2.00	26.56	6.11	32.44	2.00	2.33	1.00	1.00
M 35-1 X ICSV 25019	4.00	3.33	2.67	2.33	1.33	2.00	44.00	40.33	55.33	49.78	1.67	2.33	1.00	1.00
M 35-1 X PS 35805	2.67	3.33	1.67	2.00	1.33	2.33	42.22	55.61	54.22	73.78	1.33	3.00	1.00	1.00
M 35-1 X IS 2123	2.33	3.33	1.33	1.67	1.33	2.00	47.56	36.89	58.11	51.67	1.00	2.00	1.00	1.00
M 35-1 X IS 2146	2.67	3.67	1.67	1.67	1.33	2.33	64.56	71.78	94.56	88.00	1.00	2.00	1.00	1.00
M 35-1 X IS 18551	2.67	3.00	1.33	2.67	1.67	2.00	60.67	94.83	85.78	103.06	1.00	1.33	1.00	1.00
M 35-1 X Swarna	1.67	3.33	1.33	2.33	1.33	2.00	47.67	58.17	72.44	80.89	1.67	2.00	1.00	1.33
CSV 15 X ICSV 25019	4.33	5.00	2.00	2.00	2.00	2.33	0.00	0.00	0.78	0.00	3.00	2.67	2.00	2.00
CSV 15 X PS 35805	4.33	4.00	2.33	2.33	2.67	2.33	0.00	0.00	0.00	0.00	2.67	3.00	2.00	2.00
CSV 15 X IS 2123	4.33	4.67	2.00	2.00	1.67	2.67	6.44	3.89	12.22	7.78	2.33	2.33	1.00	1.00
CSV 15 X IS 2146	4.00	4.00	2.33	2.00	1.67	3.00	34.89	25.00	49.67	44.22	2.33	2.00	1.00	1.00
CSV 15 X IS 18551	4.67	4.00	2.67	2.33	1.33	2.00	36.44	21.00	54.44	28.83	1.67	2.00	1.00	1.00
CSV 15 X Swarna	4.33	4.67	2.67	2.67	1.33	3.00	3.78	0.00	6.22	0.00	2.67	3.00	1.00	1.00
ICSV 25019 X PS 35805	3.00	3.33	1.00	2.67	1.33	2.33	37.89	68.56	57.00	59.61	3.00	3.00	2.00	2.00
ICSV 25019 X IS 2123	3.33	3.67	2.00	2.67	1.00	2.67	44.89	47.22	58.33	61.39	1.33	2.33	1.00	1.00
ICSV 25019 X IS 2146	3.67	4.00	1.67	2.00	1.33	2.00	75.67	80.78	100.44	96.78	1.33	2.00	1.00	1.00
ICSV 25019 X IS 18551	2.33	3.67	1.00	2.00	1.33	2.00	67.00	92.44	94.11	110.11	1.00	2.00	1.00	1.00
ICSV 25019 X Swarna	4.33	5.00	2.67	2.67	2.00	2.00	17.89	8.44	29.22	13.89	3.00	3.00	1.00	1.00
PS 35805 X IS 2123	2.33	2.67	1.33	2.00	1.33	2.00	44.78	52.67	58.56	64.44	1.00	2.00	1.00	1.00
PS 35805 X IS 2146	2.33	2.67	1.67	1.67	1.33	2.33	69.33	43.78	87.67	70.33	1.00	2.33	1.00	1.00
PS 35805 X IS 18551	3.33	3.00	2.00	1.33	2.00	2.00	62.22	55.00	89.22	83.67	1.00	2.00	1.00	1.00
PS 35805 X Swarna	3.33	4.67	2.00	2.67	2.33	2.33	28.67	11.67	44.67	9.89	3.00	3.00	1.00	1.00
IS 2123 X IS 2146	3.33	3.33	1.33	2.33	1.67	2.00	46.44	77.33	70.67	81.67	1.00	2.00	1.00	1.00
IS 2123 X IS 18551	2.00	2.33	1.00	1.33	1.00	2.00	55.22	69.39	85.11	79.78	1.00	1.00	1.00	1.00
IS 2123 X Swarna	3.33	3.67	2.33	2.00	1.33	2.00	24.11	30.33	43.00	32.44	1.67	2.33	1.00	1.00
IS 2146 X IS 18551	1.67	2.00	1.00	1.00	1.33	2.00	88.33	104.33	124.67	115.00	1.33	1.00	1.00	1.00
IS 2146 X Swarna	3.67	4.33	1.67	2.33	1.33	2.00	83.11	46.06	112.11	56.00	2.00	2.67	1.00	1.00
IS 18551 X Swarna	3.33	4.67	2.00	2.33	1.33	2.33	59.56	56.89	87.00	84.11	1.67	2.00	1.00	1.00
**RECIPROCAL CROSSES**														
Phule Anuradha X ICSV 700	3.00	2.67	1.33	2.00	1.33	2.00	72.78	74.22	105.00	93.00	1.33	2.00	1.00	1.00
M 35-1 X ICSV 700	3.67	3.33	2.33	2.33	1.67	2.00	48.78	64.78	77.78	82.44	1.33	2.00	1.00	1.00
M 35-1 X Phule Anuradha	2.33	4.00	1.33	2.00	1.00	2.33	53.89	41.78	69.22	46.67	1.00	2.00	1.00	1.00
CSV 15 X ICSV 700	4.00	5.00	2.33	2.00	1.67	2.33	20.56	4.33	39.67	13.17	1.67	2.33	2.00	2.00
CSV 15 X Phule Anuradha	3.67	4.33	1.67	2.00	2.67	2.33	3.22	0.00	4.11	0.00	1.67	2.33	1.00	1.00
CSV 15 X M 35-1	3.67	5.00	2.00	2.33	1.33	2.67	5.89	0.00	8.33	0.00	1.67	2.33	1.00	1.00
ICSV 25019 X ICSV 700	2.67	2.67	1.33	1.33	1.67	2.00	58.56	70.22	79.89	76.00	1.67	2.33	2.00	2.00
ICSV 25019 X Phule Anuradha	3.00	3.67	2.00	1.67	1.67	2.33	40.67	72.39	51.89	68.06	1.33	2.33	1.00	1.00
ICSV 25019 X M 35-1	3.67	3.33	2.00	2.33	1.00	2.00	48.78	52.17	57.89	53.61	2.00	2.00	1.00	1.00
ICSV 25019 X CSV 15	3.33	4.67	1.67	2.33	1.67	2.67	4.33	0.00	3.67	0.00	3.00	3.00	2.00	2.00
PS 35805 X ICSV 700	3.00	3.67	1.67	2.00	2.00	2.00	45.22	62.39	79.44	71.94	1.67	2.33	2.00	2.00
PS 35805 X Phule Anuradha	1.67	3.00	1.00	1.67	1.67	2.00	48.00	58.67	65.78	60.11	1.67	2.00	1.00	1.00
PS 35805 X M 35-1	2.33	4.00	1.33	2.33	1.33	2.33	41.00	42.89	60.78	54.61	1.67	2.33	1.00	1.00
PS 35805 X CSV 15	2.67	4.33	1.00	2.00	1.83	2.67	5.78	29.94	12.22	36.39	2.67	3.00	1.67	1.67
PS 35805 X ICSV 25019	2.33	3.67	1.67	2.00	1.67	2.00	42.22	68.61	59.00	64.00	3.00	3.00	2.00	2.00
IS 2123 X ICSV 700	1.67	2.33	1.33	1.33	1.33	1.67	45.78	68.89	71.00	70.33	1.00	2.00	1.00	1.00
IS 2123 X Phule Anuradha	3.67	4.00	1.67	2.33	1.33	2.33	46.83	45.17	59.22	47.78	1.00	2.00	1.00	1.00
IS 2123 X M 35-1	3.67	3.33	1.67	1.67	1.33	2.00	40.11	50.33	60.33	59.00	1.00	2.00	1.00	1.00
IS 2123 X CSV 15	4.67	5.00	2.67	2.33	2.00	2.33	1.00	6.72	7.78	5.44	2.00	2.33	1.00	1.00
IS 2123 X ICSV 25019	2.67	3.33	1.33	1.33	1.00	2.00	31.89	41.39	47.67	51.83	1.00	2.33	1.00	1.00
IS 2123 X PS 35805	3.00	3.00	1.33	1.67	1.33	1.67	63.56	63.44	79.44	60.44	1.33	2.00	1.00	1.00
IS 2146 X ICSV 700	3.33	2.67	1.33	1.33	1.33	2.00	79.33	60.22	110.78	70.89	1.00	2.00	1.00	1.00
IS 2146 X Phule Anuradha	2.33	3.67	1.67	2.00	2.00	2.33	76.00	87.33	109.56	86.78	1.67	2.00	1.00	1.00
IS 2146 X M 35-1	2.33	3.00	1.00	1.33	1.67	2.00	81.39	88.56	85.50	99.44	1.00	2.00	1.00	1.00
IS 2146 X CSV 15	4.00	4.33	2.33	2.33	1.67	2.33	35.89	4.33	53.78	11.33	2.00	2.33	1.00	1.00
IS 2146 X ICSV 25019	2.50	3.33	1.00	2.00	1.00	1.67	95.78	78.11	113.00	94.22	1.00	2.00	1.00	1.00
IS 2146 X PS 35805	2.67	2.67	1.67	1.33	1.33	2.00	91.78	63.56	118.67	66.67	1.33	2.00	1.00	1.00
IS 2146 X IS 2123	2.00	2.00	1.00	1.00	1.67	1.67	90.00	91.33	106.00	110.78	1.00	1.67	1.00	1.00
IS 18551 X ICSV 700	2.67	2.00	1.00	1.00	1.00	1.33	77.89	60.44	101.56	78.78	1.00	1.67	1.00	1.00
IS 18551 X Phule Anuradha	3.00	3.00	1.67	2.00	1.33	2.00	61.78	58.33	95.00	76.89	1.00	1.67	1.00	1.00
IS 18551 X M 35-1	2.67	3.33	1.00	1.67	1.00	2.00	57.33	69.33	83.22	94.22	1.00	1.33	1.00	1.00
IS 18551 X CSV 15	3.67	4.33	2.33	2.33	1.67	2.00	39.00	38.11	67.33	59.39	1.67	1.67	1.00	1.00
IS 18551 X ICSV 25019	3.33	3.00	1.67	1.67	1.67	2.00	81.78	73.00	107.33	77.56	1.00	1.67	1.00	1.00
IS 18551 X PS 35805	2.00	2.33	1.33	2.00	1.00	2.00	64.56	86.22	99.00	96.78	1.00	1.67	1.00	1.00
IS 18551 X IS 2123	2.33	2.00	1.33	1.33	1.00	2.00	70.11	75.00	99.11	93.78	1.00	1.67	1.00	1.00
IS 18551 X IS 2146	2.33	1.67	1.33	1.00	1.67	2.00	89.33	94.44	112.11	107.11	1.00	1.67	1.00	1.00
Swarna X ICSV 700	4.33	4.67	2.33	2.00	1.67	2.00	66.89	58.94	108.89	74.67	2.33	2.00	1.00	1.00
Swarna X Phule Anuradha	3.67	4.33	2.33	2.00	1.67	2.00	16.11	20.89	26.67	32.89	2.33	2.33	1.00	1.00
Swarna X M 35-1	4.33	5.00	2.00	2.67	2.33	2.33	21.89	22.33	26.89	17.89	2.33	2.00	1.00	1.00
Swarna X CSV 15	4.33	5.00	2.67	2.67	2.33	2.33	4.78	0.00	9.78	0.00	2.67	3.00	1.00	1.00
Swarna X ICSV 25019	3.67	4.67	2.67	3.00	1.67	2.00	13.22	2.11	15.89	3.22	3.00	3.00	1.00	1.00
Swarna X PS 35805	4.33	4.67	2.67	2.00	1.33	2.67	16.00	5.44	19.33	5.39	3.00	3.00	1.00	1.00
Swarna X IS 2123	4.67	4.67	2.67	3.00	1.67	2.00	16.89	21.56	29.56	19.44	2.00	2.67	1.00	1.00
Swarna X IS 2146	4.67	4.33	1.67	2.33	1.33	2.00	67.22	55.28	92.78	73.67	2.67	3.00	1.00	1.00
Swarna X IS 18551	3.67	4.67	2.33	2.33	2.00	2.00	50.22	78.22	89.78	97.44	2.33	2.33	1.00	1.00
Mean	3.17	3.52	1.74	1.96	1.55	2.12	49.70	50.90	68.70	60.45	1.70	2.19	1.20	1.16
SE ±	0.57	0.35	0.40	0.34	0.36	0.24	7.69	10.22	9.06	9.92	0.24	0.23	0.03	0.05
Vr (99, 198)	2.04^**^	8.03^**^	1.70^**^	2.18^**^	1.08^*NS*^	1.84^**^	12.46^**^	7.75^**^	14.81^**^	11.12^**^	8.07^**^	4.74^**^	118.09^**^	58.48^**^
LSD (P 0.05)	1.58	0.97	1.12	0.96	0.99	0.68	21.45	28.50	25.26	27.65	0.68	0.65	0.09	0.13

** *F probability significant at P = 0.01; NS, non-significant F-value; R, rainy season; PR, postrainy season*.

### Combining ability analysis

#### Analysis of variance for combining ability

The estimation of mean sum of squares (ANOVA) for general combining ability (GCA) of parents, and specific combining ability (SCA) of the hybrids showed significant (*P* ≤ 0.01) general combining ability effects for all the traits studied in the postrainy season, and for most of the traits in the rainy season (Table 3). The mean sum of squares due to SCA were significant for most of the traits during the rainy and postrainy seasons, with the exception of leaf glossy score and plants with shoot fly eggs during the rainy season, number of shoot fly eggs/plant and leafsheath pigmentation across seasons, and waxy bloom in the postrainy season. The mean sum of squares for the reciprocal crosses were significant for trichome density on abaxial and adaxial leaf surfaces across seasons; overall resistance score during the rainy season, and plants with shoot fly eggs, and leaf glossy score during the postrainy season, suggesting influence of the cytoplasmic factors in expression of the traits associated with resistance to shoot fly in sorghum.

**Table 3:** **Analysis of variance (ANOVA) showing mean sum of squares of general, specific, and reciprocal combining abilities of F_**1**_(10 × 10) diallel (ICRISAT, Patancheru, 2013–14)**.

**Source**	**GCA**		**SCA**		**Reciprocal**		**Error**	

	**2013 R**	**2013 PR**	**2013 R**	**2013 PR**	**2013 R**	**2013 PR**	**2013 R**	**2013 PR**
Plants with shoot fly eggs (%)	62.20	2707.91^**^	42.10	524.18^**^	56.02	256.12^**^	40.36	110.81
Number of shoot fly eggs/plant	0.20	0.33^**^	0.19	0.09	0.23	0.06	0.18	0.07
Shoot fly deadhearts (%)	673.87^**^	2410.48^**^	190.33^**^	146.67	136.37	93.05	101.81	107.64
Overall resistance score	3.14^**^	3.23^**^	0.89^**^	0.77^**^	0.448^*^	0.51	0.29	0.38
Leaf glossy score	2.89^**^	7.34^**^	0.43	0.49^**^	0.42	0.17^*^	0.32	0.12
Plant vigor score	1.13^**^	1.33^**^	0.24^*^	0.17^*^	0.13	0.13	0.16	0.12
Leafsheath pigmentation	0.34	0.65^**^	0.11	0.06	0.12	0.05	0.13	0.06
Trichome density on abaxial leaf surface	6126.24^**^	6846.39^**^	292.89^**^	236.67^**^	104.26^**^	173.84^**^	59.16	104.42
Trichome density on adaxial leaf surface	10789.18^**^	9028.91^**^	346.52^**^	314.57^**^	166.63^**^	285.76^**^	81.99	98.31
Plant color	0.82^**^	0.80^**^	0.12^**^	0.13^**^	0.00	0.00	0.00	0.00
Waxy bloom	3.91^**^	2.22^**^	0.22^**^	0.07	0.05	0.05	0.06	0.05

*, ** *F probability significant at P= 0.05 and 0.01, respectively; GCA, general combining ability; SCA, specific combining ability; R, rainy season; PR, postrainy season*.

### Estimates of *gca, sca*, and reciprocal effects of parents and hybrids

#### General combining ability (gca) effects

##### gca effects of shoot fly resistance traits

The general combining ability (*gca*) effects for shoot fly deadhearts ranged from –8.13 (PS 35805) to 9.80 (CSV 15) in the rainy season, and from –8.80 (PS 35805) to 20.86 (CSV 15) in the postrainy season (Table 4). The genotypes Phule Anuradha, ICSV 25019, PS 35805, IS 2123, and IS 18551 exhibited negative and significant *gca* effects, with a few exceptions. M 35-1 and Phule Anuradha exhibited positive but non-significant *gca* effects in the rainy season but showed negative *gca* effects in the postrainy season. CSV 15 and Swarna exhibited significant positive *gca* effects across seasons. Similar pattern was observed in case of overall resistance score across seasons.

**Table 4:** **Estimates of general combining ability effects of shoot fly resistance and morphological traits of parents (10 × 10 diallel) across seasons (ICRISAT, Patancheru, 2013–14)**.

**Traits**	**ICSV 700**	**Phule Anuradha**	**M 35-1**	**CSV 15**	**ICSV 25019**	**PS 35805**	**IS 2123**	**IS 2146**	**IS 18551**	**Swarna**
Plants with shoot fly eggs (%)	(−0.71)	(−5.25)^*^	(−6.11)^**^	(20.65)^**^	(−4.58)^*^	(−6.91)^**^	(−7.54)^**^	(−4.89)^*^	(−7.50)^**^	(22.83)^**^
Number of shoot fly eggs/plant	(0.02)	(−0.16)^**^	(−0.09)	(0.16)^**^	(−0.07)	(−0.13)^*^	(−0.02)	(0.01)	(0.02)	(0.26)^**^
Shoot fly deadhearts (%)	−0.45 (−5.25)^*^	1.88 (−4.51)^*^	2.4 (−4.15)	9.80^**^ (20.86)^**^	−6.22^**^ (−3.28)	−8.13^**^ (−8.80)^**^	−4.91* (−6.26)^**^	−0.32 (−2.81)	−2.07 (−6.10)^**^	8.03^**^ (20.30)^**^
ORS	−0.06 (0.38)^**^	−0.04 (−0.03)	0.25* (−0.2)	0.58^**^ (0.43)^**^	−0.45^**^ (−0.08)	−0.44^**^ (−0.03)	−0.24* (−0.43)^**^	−0.23* (−0.47)^**^	−0.09 (−0.32)^*^	0.71^**^ (0.75)^**^
Leaf glossy score	−0.14 (−0.53)^**^	−0.22 (−0.05)	−0.11 (0.05)	0.73^**^ (0.98)^**^	−0.03 (0.13)	−0.22 (−0.05)	−0.02 (−0.45)^**^	−0.30* (−0.47)^**^	−0.34^**^ (−0.68)^**^	0.66^**^ (1.07)^**^
Plant vigor score	−0.15 (−0.21)^**^	−0.13 (−0.11)	−0.01 (0.1)	0.41^**^ (0.34)^**^	−0.08 (0.14)	−0.08 (0.07)	−0.01 (−0.16)^*^	−0.24^**^ (−0.28)^**^	−0.17* (−0.31)^**^	0.45^**^ (0.44)^**^
Leafsheath pigmentation	(−0.27)^**^	(0.09)	(0.04)	(0.38)^**^	(−0.01)	(0.06)	(−0.07)	(−0.06)	(−0.22)^**^	(0.06)
Trichome density on abaxial leaf surface	16.52^**^ (14.75)^**^	2.66 (2.82)	−0.07 (4.06)	−35.24^**^ (−38.35)^**^	−3.93* (0.24)	−2.81 (−1.09)	−5.36^**^ (−0.12)	26.18^**^ (18.19)^**^	16.76^**^ (22.51)^**^	−14.72^**^ (−23.01)^**^
Trichome density on adaxial leaf surface	29.09^**^ (20.90)^**^	2.27 (0.32)	−3.48 (3.54)	−45.04^**^ (−41.49)^**^	−6.56^**^ (−3.95)	−4.88* (−2.85)	−8.36^**^ (−1.92)	30.03^**^ (21.07)^**^	24.69^**^ (28.75)^**^	−17.76^**^ (−24.38)^**^
Waxy bloom	−0.32^**^ (−0.12*)	−0.20^**^ (−0.14)^**^	−0.24^**^ (−0.12)^*^	0.58^**^ (0.35)^**^	0.33^**^ (0.28)^**^	0.28^**^ (0.31)^**^	−0.44^**^ (−0.17)^**^	−0.29^**^ (−0.19)^**^	−0.45^**^ (−0.64)^**^	0.75^**^ (0.43)^**^
Plant color	0.24^**^ (0.24)^**^	−0.16^**^ (−0.16)^**^	−0.16^**^ (−0.14)^**^	0.23^**^ (0.22)^**^	0.24^**^ (0.24)^**^	0.23^**^ (0.22)^**^	−0.16^**^ (−0.16)^**^	−0.16^**^ (−0.16)^**^	−0.16^**^ (−0.16)^**^	−0.16^**^ (−0.14)^**^

*, ** *t-test significant at P = 0.05 and 0.01, respectively; ORS, overall resistance score; The values inside the parentheses are for postrainy season and outside the parentheses for rainy season*.

The genotypes CSV 15 and Swarna showed significant and positive *gca* effects for percentage plants with shoot fly eggs and number of shoot fly eggs/plant in the postrainy season (Table 4). Phule Anuradha, M 35-1, ICSV 25019, PS 35805, IS 2123, IS 2146, and IS 18551 exhibited significant negative *gca* effects for plants with shoot fly eggs (%), whereas Phule Anuradha and PS 35805 exhibited significant negative *gca* effects for number of shoot fly eggs/plant in the postrainy season.

##### gca effects of morphological traits

The *gca* effects of leaf glossy score ranged from –0.34 (IS 18551) to 0.73 (CSV 15), and for plant vigor score from –0.24 (IS 2146) to 0.45 (Swarna) in the rainy season. In the postrainy season the *gca* effects for leaf glossy score ranged from –0.68 (IS 18551) to 1.07 (Swarna), for plant vigor score from –0.31 (IS 18551) to 0.44 (Swarna) and for leafsheath pigmentation from –0.27 (ICSV 700) to 0.38 (CSV 15) in the postrainy season (Table 4). IS 2146 and IS 18551 exhibited significant and negative *gca* effects, and CSV 15 and Swarna exhibited significant and positive *gca* effects for leaf glossy score and for plant vigor score across seasons. ICSV 700 and IS 2123 exhibited significant negative *gca* effects in the postrainy season, both for leaf glossy score and plant vigor score. ICSV 700 and IS 18551 exhibited significant negative and CSV 15 significant positive *gca* effects for leafsheath pigmentation in the postrainy season.

The general combining ability effects for trichome density on the abaxial leaf surface ranged from –35.24 (CSV 15) to 16.52 (ICSV 700) in the rainy season; and from –38.35 (CSV 15) to 22.51 (IS 18551) in the postrainy season (Table 4). The *gca* effects of trichome density on the adaxial leaf surface ranged from –45.04 (CSV 15) to 30.03 (IS 2146) in the rainy season, and from –41.49 (CSV 15) to 28.75 (IS 18551) in the postrainy season. CSV 15, ICSV 25019, IS 2123, PS 35805, and Swarna exhibited significant negative *gca* effects, while ICSV 700, IS 2146, and IS 18551 exhibited significant positive *gca* effects in the rainy season and CSV 15 and Swarna showed significant negative, and ICSV 700, IS 2146, and IS 18551 significant positive *gca* effects for trichome density on abaxial and adaxial leaf surfaces in the postrainy season.

The *gca* effects of waxy bloom ranged from -0.45 (IS 18551) to 0.75 (Swarna) in the rainy season, and from –0.64 (IS 18551) to 0.43 (Swarna) in the postrainy season (Table 4). ICSV 700, Phule Anuradha, M 35-1, IS 2123, IS 2146, and IS 18551 exhibited significant and negative *gca* effects; while CSV 15, ICSV 25019, PS 35805, and Swarna exhibited significant and positive *gca* effects across seasons. The general combining ability effect of plant color ranged from –0.16 to 0.24 in the rainy season, and –0.14 to 0.24 in the postrainy season.

### Specific combining ability (*sca*) effects

#### *sca* effects of shoot fly resistance traits

The *sca* effects of plants with shoot fly eggs (%) ranged from –13.04 to 37.56 in the postrainy season (Table 5). The range of *sca* effects for shoot fly deadhearts (%) varied from –12.42 to 17.74 in the rainy season, and the overall resistance score from –1.16 to 1.15 in the rainy season, and from –0.80 to 1.22 in the postrainy season.

**Table 5:** **Estimates of specific combining ability effects of shoot fly resistance traits of F_**1**_ crosses (10 × 10 diallel) of sorghum, across seasons (ICRISAT, Patancheru, 2013-14)**.

**Pedigree**	**Plants with shoot fly eggs (%)**	**Shoot fly deadhearts (%)**	**ORS**	

	**2013 PR**	**2013 R**	**2013 R**	**2013 PR**
ICSV 700 X Phule Anuradha	-7.39	2.06	-0.83^*^	-0.07
ICSV 700 X M 35-1	5.46	7.48	-0.61	0.43
ICSV 700 X CSV 15	9.82	-3.05	-0.28	0.13
ICSV 700 X ICSV 25019	-8.03	1.91	-0.09	-0.35
ICSV 700 X PS 35805	-1.82	-7.51	-0.43	-0.40
ICSV 700 X IS 2123	6.69	-0.05	0.71^*^	0.50
ICSV 700 X IS 2146	-0.92	4.78	0.86^*^	-0.80^*^
ICSV 700 X IS 18551	6.71	-1.08	0.56	0.88^*^
ICSV 700 X Swarna	19.38^**^	8.12	-0.41	-0.35
Phule Anuradha X M 35-1	-13.04	-0.99	0.37	0.35
Phule Anuradha X CSV 15	-1.50	1.30	-0.46	-0.12
Phule Anuradha X ICSV 25019	11.93	-2.22	0.40	0.23
Phule Anuradha X PS 35805	-1.87	0.07	0.56	-0.32
Phule Anuradha X IS 2123	9.71	6.05	0.69	-0.75
Phule Anuradha X IS 2146	12.64	10.14	-0.15	0.45
Phule Anuradha X IS 18551	-3.43	-10.91	0.21	0.30
Phule Anuradha X Swarna	13.82^*^	2.33	0.07	-0.60
M 35-1 X CSV 15	6.02	2.14	-0.58	-0.28
M 35-1 X ICSV 25019	-5.02	15.29^*^	-0.55	0.07
M 35-1 X PS 35805	3.66	8.96	0.44	-0.65
M 35-1 X IS 2123	10.48	-1.88	-0.26	0.75
M 35-1 X IS 2146	0.06	-11.96	0.23	0.45
M 35-1 X IS 18551	12.27	-12.42	0.26	0.47
M 35-1 X Swarna	-0.83	-5.55	-1.21^**^	-0.10
CSV 15 X ICSV 25019	37.56^**^	6.55	0.61	0.93^*^
CSV 15 X PS 35805	7.01	4.18	0.44	1.22^**^
CSV 15 X IS 2123	-2.72	5.34	-0.43	-0.38
CSV 15 X IS 2146	18.93^**^	-2.56	-0.77^*^	-0.68
CSV 15 X IS 18551	2.09	6.84	-0.08	-0.17
CSV 15 X Swarna	-4.88	-4.48	0.62	-0.23
ICSV 25019 X PS 35805	1.37	-5.52	0.96^**^	0.40
ICSV 25019 X IS 2123	-5.73	-5.53	-0.90^*^	0.13
ICSV 25019 X IS 2146	-4.60	11.52	-1.16^**^	-0.67
ICSV 25019 X IS 18551	-11.08	17.74^**^	-0.39	-0.32
ICSV 25019 X Swarna	5.75	6.37	1.15^**^	0.95^*^
PS 35805 X IS 2123	-1.57	8.66	-0.58	0.42
PS 35805 X IS 2146	1.76	7.80	-0.09	0.45
PS 35805 X IS 18551	-0.24	3.17	-0.73^*^	0.13
PS 35805 X Swarna	9.26	-0.70	0.14	0.57
IS 2123 X IS 2146	-7.87	-4.62	0.71^*^	0.52
IS 2123 X IS 18551	-3.39	1.72	0.24	0.20
IS 2123 X Swarna	10.70	0.41	-0.73^*^	-0.37
IS 2146 X IS 18551	-4.04	3.57	0.23	0.23
IS 2146 X Swarna	5.55	6.09	-0.40	-0.33
IS 18551 X Swarna	17.53^*^	7.02	-0.55	-0.48

*, ** *t-test significant at P = 0.05 and 0.01, respectively; ORS, overall resistance score; R, rainy season; PR, postrainy season*.

ICSV 700 X Swarna, Phule Anuradha X Swarna, CSV 15 X ICSV 25019, CSV 15 X IS 2146, and IS 18551 X Swarna exhibited significant positive *sca* effects for percentage plants with shoot fly eggs in the postrainy season. M 35-1 X ICSV 25019 and ICSV 25019 X IS 18551 exhibited significant positive *sca* effects for shoot fly deadhearts (%) in the rainy season (Table 5). ICSV 700 X IS 2123, ICSV 700 X IS 2146, ICSV 25019 X PS 35805, ICSV 25019 X Swarna, and IS 2123 X IS 2146 in the rainy season; and ICSV 700 X IS 18551, CSV 15 X ICSV 25019, CSV 15 X PS 35805, and ICSV 25019 X Swarna in the postrainy season exhibited significant positive *sca* effects for overall resistance score. ICSV 700 X Phule Anuradha, M 35-1 X Swarna, CSV 15 X IS 2146, ICSV 25019 X IS 2123, ICSV 25019 X IS 2146, PS 35805 X IS 18551, and IS 2123 X Swarna in the rainy season; and ICSV 700 X IS 2146 in the postrainy season exhibited significant negative *sca* effects for overall resistance score.

#### *sca* effects of morphological traits

The *sca* effects of leaf glossy score ranged from –0.73 to 0.90 (in the postrainy season), for plant vigor score from –0.52 to 0.68 and –0.55 to 0.48, for trichome density on the abaxial leaf surface –25.68 to 15.47 and –22.85 to 18.53, for trichome density on the adaxial leaf surface from –25.92 to 26.80 and –29.98 to 25.95, and for waxy bloom from –0.55 to 0.72 (in the rainy season only) respectively, in the rainy and postrainy seasons (Table 6).

**Table 6:** **Estimates of specific combining ability effects of morphological traits of F_**1**_ crosses (10 × 10 diallel) of sorghum across seasons (ICRISAT, Patancheru, 2013-14)**.

**Pedigree**	**Leaf glossy score**	**Plant vigor score**		**Trichome density on abaxial leaf surface**		**Trichome density on adaxial leaf surface**		**Waxy bloom**
	**2013 PR**	**2013 R**	**2013 PR**	**2013 R**	**2013 PR**	**2013 R**	**2013 PR**	**2013 R**
ICSV 700 X Phule Anuradha	-0.43	0.20	0.20	-2.70	-1.84	-1.38	0.89	0.02
ICSV 700 X M 35-1	0.30	0.25	0.48^*^	-8.57	4.22	-1.09	8.72	0.05
ICSV 700 X CSV 15	0.20	0.33	-0.09	5.10	2.24	11.59	17.36^**^	-0.26
ICSV 700 X ICSV 25019	-0.45^*^	-0.02	-0.55^*^	1.11	7.16	4.85	11.93	-0.18
ICSV 700 X PS 35805	0.90^**^	-0.02	0.35	-16.06^**^	-7.21	-15.78^**^	-10.75	-0.13
ICSV 700 X IS 2123	0.13	-0.08	0.08	-13.41^**^	0.96	-14.49^*^	-0.38	0.09
ICSV 700 X IS 2146	0.32	-0.18	0.36	-25.68^**^	-18.01^**^	-25.92^**^	-29.98^**^	0.10
ICSV 700 X IS 18551	-0.13	-0.25	-0.27	-11.53^*^	-12.56	-20.37^**^	-22.50^**^	0.10
ICSV 700 X Swarna	0.62^**^	-0.13	-0.02	15.47^**^	15.88^*^	26.80^**^	17.75^**^	-0.10
Phule Anuradha X M 35-1	0.15	-0.28	0.21	0.29	-7.61	1.19	-4.86	-0.23
Phule Anuradha X CSV 15	-0.28	-0.19	-0.35	1.58	2.88	0.14	0.20	-0.38^*^
Phule Anuradha X ICSV 25019	-0.10	0.13	-0.15	-2.08	10.24	-3.69	5.54	-0.46^**^
Phule Anuradha X PS 35805	-0.08	-0.21	-0.09	2.43	3.26	0.93	4.42	-0.25
Phule Anuradha X IS 2123	0.48^*^	0.23	0.48^*^	-1.13	2.54	-2.22	-1.84	-0.03
Phule Anuradha X IS 2146	0.67^**^	0.46	0.26	0.04	6.54	2.87	5.67	0.49^**^
Phule Anuradha X IS 18551	0.38	-0.11	0.13	-0.77	-6.34	-0.55	-2.07	0.32^*^
Phule Anuradha X Swarna	-0.03	0.27	-0.29	-15.98^**^	-15.68^*^	-22.27^**^	-10.86	0.12
M 35-1 X CSV 15	0.12	-0.14	0.10	-10.49^*^	-3.34	-12.94^*^	-6.28	-0.18
M 35-1 X ICSV 25019	-0.37	0.68^*^	0.13	0.67	-8.95	-2.05	-8.35	0.07
M 35-1 X PS 35805	0.15	-0.16	0.03	-5.22	-4.63	-2.83	3.05	-0.21
M 35-1 X IS 2123	0.22	-0.23	-0.24	-0.45	-11.24	2.40	-6.74	0.00
M 35-1 X IS 2146	0.23	-0.16	-0.29	-2.86	7.02	-5.20	8.66	-0.15
M 35-1 X IS 18551	0.28	-0.39	0.41	-7.39	4.60	-5.41	5.89	0.02
M 35-1 X Swarna	-0.47^*^	-0.52^*^	0.00	-0.16	8.30	2.24	9.77	-0.18
CSV 15 X ICSV 25019	0.20	-0.24	-0.27	-8.40	-12.79	-14.88^*^	-15.02^*^	0.42^**^
CSV 15 X PS 35805	-0.28	-0.41	-0.20	-8.80	3.50	-12.68^*^	2.09	0.14
CSV 15 X IS 2123	0.78^**^	0.19	0.03	-5.41	-7.13	-5.31	-10.43	0.35^*^
CSV 15 X IS 2146	0.13	0.43	0.15	-5.29	-16.08^*^	-1.97	-12.25	0.20
CSV 15 X IS 18551	0.35	0.53^*^	0.35	6.46	-5.52	12.54^*^	-3.61	-0.13
CSV 15 X Swarna	-0.73^**^	0.07	-0.07	4.51	10.45	2.12	5.42	-0.33^*^
ICSV 25019 X PS 35805	-0.10	-0.26	0.16	-2.93	18.53^**^	0.73	8.15	0.72^**^
ICSV 25019 X IS 2123	0.30	0.01	0.06	-2.06	-6.71	-0.78	2.03	-0.40^*^
ICSV 25019 X IS 2146	0.48^*^	-0.09	0.18	13.72^**^	10.12	14.57^*^	17.93^**^	-0.55^**^
ICSV 25019 X IS 18551	0.37	-0.16	0.05	11.83^*^	9.07	13.89^*^	8.58	-0.55^**^
ICSV 25019 X Swarna	0.12	0.55^*^	0.30	-15.52^**^	-22.85^**^	-21.82^**^	-23.58^**^	0.25
PS 35805 X IS 2123	-0.18	-0.33	-0.04	12.61^*^	8.36	13.54^*^	6.77	-0.35^*^
PS 35805 X IS 2146	-0.33	0.24	-0.25	7.46	-14.33^*^	9.32	-10.17	-0.49^**^
PS 35805 X IS 18551	-0.12	0.18	-0.05	-0.29	-1.72	5.63	3.87	-0.49^**^
PS 35805 X Swarna	0.13	0.22	-0.14	-9.86	-18.25^**^	-14.07^*^	-25.58^**^	0.30
IS 2123 X IS 2146	0.07	-0.33	0.15	-2.33	15.36^*^	-2.03	16.63^*^	0.05
IS 2123 X IS 18551	-0.22	-0.39	-0.15	1.54	-1.10	7.08	-0.51	0.22
IS 2123 X Swarna	0.03	0.32	0.26	-9.15	-1.83	-6.31	-8.21	-0.15
IS 2146 X IS 18551	-0.53^*^	-0.16	-0.37	-3.84	7.79	-5.03	0.79	0.24
IS 2146 X Swarna	0.22	-0.28	0.21	13.97^**^	4.59	21.47^**^	7.69	0.20
IS 18551 X Swarna	0.77^**^	0.15	0.25	3.12	17.15^*^	12.76^*^	25.95^**^	0.04

*, ** *t-test significant at P = 0.05 and 0.01, respectively; R, rainy season; PR, postrainy season*.

ICSV 700 X PS 35805, ICSV 700 X Swarna, Phule Anuradha X IS 2123, Phule Anuradha X IS 2146, CSV 15 X IS 2123, ICSV 25019 X IS 2146, and IS 18551 X Swarna exhibited significant positive *sca* effects for leaf glossy score in the postrainy season (Table 6). ICSV 700 X ICSV 25019, M 35-1 X Swarna, CSV 15 X Swarna, and IS 2146 X IS 18551 exhibited significant negative *sca* effects for leaf glossy score in the postrainy season. M 35-1 X ICSV 25019, CSV 15 X IS 18551, and ICSV 25019 X Swarna in the rainy season; and ICSV 700 X M 35-1, Phule Anuradha X IS 2123 in the postrainy season exhibited significant positive *sca* effects for plant vigor score. M 35-1 X Swarna in the rainy season, and ICSV 700 X ICSV 25019 in the postrainy season exhibited negative *sca* effects for plant vigor score.

ICSV 700 X PS 35805, ICSV 700 X IS 2123, ICSV 700 X IS 2146, ICSV 700 X IS 18551, Phule Anuradha X Swarna, M 35-1 X CSV 15, ICSV 25019 X Swarna in the rainy season; and ICSV 700 X IS 2146, Phule Anuradha X Swarna, CSV 15 X IS 2146, ICSV 25019 X Swarna, PS 35805 X IS 2146, and PS 35805 X Swarna in the postrainy season showed significant negative *sca* effects for trichome density on the abaxial leaf surface (Table 6). ICSV 700 X Swarna across seasons, and ICSV 25019 X IS 2146, ICSV 25019 X IS 18551, PS 35805 X IS 2123, and IS 2146 X Swarna in the rainy season, and ICSV 25019 X PS 35805, IS 2123 X IS 2146, and IS 18551 X Swarna in the postrainy season exhibited significant positive *sca* effects for trichome density on the abaxial leaf surface.

The crosses ICSV 700 X PS 35805, ICSV 700 X IS 2123, ICSV 700 X IS 2146, ICSV 700 X IS 18551, Phule Anuradha X Swarna, M 35-1 X CSV 15, CSV 15 X ICSV 25019, CSV 15 X PS 35805, ICSV 25019 X Swarna, and PS 35805 X Swarna in the rainy season; and ICSV 700 X IS 2146, ICSV 700 X IS 18551, CSV 15 X ICSV 25019, ICSV 25019 X Swarna, and PS 35805 X Swarna in the postrainy season showed significant negative *sca* effects for trichome density on the adaxial leaf surface (Table 6). ICSV 700 X Swarna, CSV 15 X IS 18551, ICSV 25019 X IS 2146, ICSV 25019 X IS 18551, PS 35805 X IS 2123, IS 2146 X Swarna, and IS 18551 X Swarna in the rainy season; and ICSV 700 X CSV 15, ICSV 700 X Swarna, ICSV 25019 X IS 2146, IS 2123 X IS 2146 and IS 18551 X Swarna in the postrainy season exhibited significant positive *sca* effects for trichome density on the adaxial leaf surface.

The *sca* effects of Phule Anuradha X CSV 15, Phule Anuradha X ICSV 25019, CSV 15 X Swarna, ICSV 25019 X IS 2123, ICSV 25019 X IS 2146, ICSV 25019 X IS 18551, PS 35805 X IS 2123, PS 35805 X IS 2146, and PS 35805 X IS 18551 in the rainy season exhibited significant negative *sca* effects for waxy bloom trait. Phule Anuradha X IS 2146, Phule Anuradha X IS 18551, CSV 15 X ICSV 25019, CSV 15 X IS 2123, ICSV 25019 X PS 35805 in the rainy season exhibited significant positive *sca* effects for waxy bloom.

### Reciprocal combining ability effects

The crosses CSV 15 X ICSV 700, ICSV 25019 X CSV 15, IS 2123 X M 35-1, IS 2123 X CSV 15, Swarna X ICSV 700, Swarna X M 35-1 exhibited significant negative reciprocal effects; while the crosses PS 35805 X CSV 15, IS 2146 X Phule Anuradha showed significant positive reciprocal effects for plants with shoot fly eggs (%) in the postrainy season (Table 7).

**Table 7:** **Estimates of reciprocal combining ability effects of reciprocal crosses (10 × 10 diallel) of sorghum across seasons (ICRISAT, Patancheru, 2013-14)**.

**Pedigree**	**Plants with shoot fly eggs (%)**	**ORS**	**Leaf glossy score**	**Trichome density on abaxial leaf surface**		**Trichome density on abaxial leaf surface**	
	**2013 PR**	**2013 R**	**2013 PR**	**2013 R**	**2013 PR**	**2013 R**	**2013 PR**
Phule Anuradha X ICSV 700	-8.73	-	-0.17	-6.55	-7.58	-6.33	-10.44
M 35-1 X ICSV 700	6.46	0.17	0.00	8.85	9.17	15.40^*^	11.17
M 35-1 X Phule Anuradha	-4.04	0.17	-0.33	-1.28	8.39	-0.57	12.78
CSV 15 X ICSV 700	-30.43^**^	0.80^*^	-0.83^**^	15.6^**^	25.22^**^	24.70^**^	44.05^**^
CSV 15 X Phule Anuradha	8.33	-	-0.17	15.5^**^	18.25^**^	21.90^**^	19.47^**^
CSV 15 X M 35-1	-3.36	0.50	-0.33	-1.93	13.28^*^	-1.10	16.22^*^
ICSV 25019 X ICSV 700	4.47	0.33	0.00	4.90	2.84	16.20^**^	13.33^*^
ICSV 25019 X Phule Anuradha	13.57	0.17	-0.17	5.72	-8.20	8.85	-5.70
ICSV 25019 X M 35-1	-11.60	-0.50	0.00	-2.37	-5.92	-1.27	-1.92
ICSV 25019 X CSV 15	-14.18^*^	0.33	0.17	-2.17	0.00	-1.45	0.00
PS 35805 X ICSV 700	5.74	-0.33	0.17	2.15	-5.03	-2.35	-4.19
PS 35805 X Phule Anuradha	13.27	0.33	0.33	4.02	-2.78	1.22	2.22
PS 35805 X M 35-1	-11.75	0.17	-0.33	0.60	6.36	-3.27	9.58
PS 35805 X CSV 15	31.59^**^	-0.50	-0.17	-2.88	-14.97^*^	-6.10	-18.19^**^
PS 35805 X ICSV 25019	-4.37	-0.33	-0.17	-2.17	-0.03	-0.98	-2.20
IS 2123 X ICSV 700	5.99	-0.33	0.33	1.72	-2.39	3.97	8.72
IS 2123 X Phule Anuradha	0.30	-0.67	-0.50^*^	-0.93	10.97	1.15	9.22
IS 2123 X M 35-1	-13.77^*^	0.33	0.00	3.72	-6.72	-1.12	-3.67
IS 2123 X CSV 15	-20.77^**^	-0.80^*^	-0.17	2.72	-1.42	2.22	1.17
IS 2123 X ICSV 25019	-11.54	0.67	0.17	6.52	2.92	5.33	4.78
IS 2123 X PS 35805	0.30	-0.33	-0.17	-9.40	-5.39	-10.43	2.00
IS 2146 X ICSV 700	8.47	0.17	0.17	-12.60^*^	5.61	-8.88	1.56
IS 2146 X Phule Anuradha	21.01^**^	-0.17	0.00	2.62	-8.89	-5.67	0.72
IS 2146 X M 35-1	3.91	-0.17	0.33	-8.42	-8.39	4.53	-5.72
IS 2146 X CSV 15	6.73	0.50	-0.17	-0.52	10.33	-2.05	16.45^*^
IS 2146 X ICSV 25019	4.19	0.58	0.33	-10.10^*^	1.33	-6.30	1.28
IS 2146 X PS 35805	-2.92	-0.17	0.00	-11.20^*^	-9.89	-15.50^*^	1.83
IS 2146 X IS 2123	1.46	0.17	0.67^**^	-21.80^**^	-7.00	-17.70^**^	-14.56^*^
IS 18551 X ICSV 700	5.83	-0.33	0.17	-6.38	15.17^*^	0.57	8.83
IS 18551 X Phule Anuradha	0.29	-0.33	0.17	6.62	11.56	0.13	10.56
IS 18551 X M 35-1	-0.14	-0.33	-0.17	1.67	12.75	1.28	4.42
IS 18551 X CSV 15	13.44	-0.33	-0.17	-1.28	-8.56	-6.45	-15.28^*^
IS 18551 X ICSV 25019	-0.78	-	0.33	-7.40	9.72	-6.62	16.28^*^
IS 18551 X PS 35805	-8.74	-0.33	0.33	-1.17	-15.61^*^	-4.90	-6.56
IS 18551 X IS 2123	2.69	-0.17	0.17	-7.43	-2.81	-7.00	-7.00
IS 18551 X IS 2146	-8.57	0.50	0.17	-0.50	4.94	6.28	3.95
Swarna X ICSV 700	-16.19^*^	1.5^**^	0.00	0.10	-0.42	-2.05	0.06
Swarna X Phule Anuradha	2.16	0.33	0.17	5.57	-5.86	4.30	-7.36
Swarna X M 35-1	-17.92^*^	-0.33	-0.83^**^	12.90^*^	17.92^**^	22.8^**^	31.50^**^
Swarna X CSV 15	-11.89	-0.17	-0.17	-0.52	0.00	-1.78	0.00
Swarna X ICSV 25019	-1.95	-	0.17	2.33	3.17	6.65	5.33
Swarna X PS 35805	-6.99	0.33	0.00	6.32	3.11	12.70^*^	2.25
Swarna X IS 2123	-5.80	-1.30^**^	-0.50^*^	3.63	4.39	6.73	6.50
Swarna X IS 2146	11.37	-0.33	0.00	7.93	-4.61	9.67	-8.83
Swarna X IS 18551	-3.00	-	0.00	4.67	-10.67	-1.42	-6.67

*, ** *t-test significant at P = 0.05 and 0.01, respectively; ORS, overall resistance score*.

IS 2123 X CSV 15 and Swarna X IS 2123 exhibited significant negative reciprocal effects; and CSV 15 X ICSV 700 and Swarna X ICSV 700 exhibited significant positive reciprocal effects for overall resistance score during the rainy season. CSV 15 X ICSV 700, IS 2123 X Phule Anuradha, Swarna X M 35-1, and Swarna X IS 2123 exhibited significant negative reciprocal effects, and IS 2146 X IS 2123 exhibited positive reciprocal effects for leaf glossy score in the postrainy season.

IS 2146 X ICSV 700, IS 2146 X ICSV 25019, IS 2146 X PS 35805, IS 2146 X IS 2123 in the rainy season, and PS 35805 X CSV 15, IS 18551 X ICSV 25019 in the postrainy season exhibited significant negative reciprocal effects for trichome density on the abaxial leaf surface (Table 7). CSV 15 X ICSV 700, CSV 15 X Phule Anuradha, and Swarna X M 35-1 across seasons, and CSV 15 X M 35-1, and IS 18551 X IS 2123 in the postrainy season exhibited significant positive reciprocal effects for trichome density on the abaxial leaf surface.

IS 2146 X PS 35805 in the rainy season, IS 2146 X IS 2123 across seasons, and IS 18551 X CSV 15 in the postrainy season exhibited significant negative reciprocal effects for trichome density on the adaxial leaf surface (Table 7). M 35-1 X ICSV 700 and Swarna X PS 35805 in the rainy season, and CSV 15 X ICSV 700, CSV 15 X Phule Anuradha, ICSV 25019 X ICSV 700, Swarna X M 35-1 across seasons, and CSV 15 X M 35-1, IS 2146 X CSV 15 and IS 18551 X ICSV 25019 in the postrainy season exhibited significant positive reciprocal effects for trichome density on the adaxial leaf surface.

### Combining ability estimates and genetic parameters of shoot fly resistance and morphological traits

Shoot fly deadhearts (%) in the rainy season, and plants with shoot fly eggs (%) in the postrainy season showed higher variance due to specific combining ability (σ^2^s) than the variance due to general combining ability (σ^2^g), indicating the predominance of dominance gene action in controlling the expression of resistance to sorghum shoot fly, *A. soccata* (Table 8). This was confirmed by higher dominance variance than the additive variance for these traits. The other traits that had σ^2^g and σ^2^s on par with each other exhibited both additive and non-additive type of gene action.

**Table 8:** **Estimates of various genetic parameters for different shoot fly resistance and morphological traits of sorghum across seasons (ICRISAT, Patancheru, 2013-14)**.

**Traits**	**Plants with shoot fly eggs (%)**	**Number of shoot fly eggs/plant**	**Shoot fly deadhearts (%)**	**ORS**	**Leaf glossy score**	**Plant vigor score**	**Leafsheath pigmentation**	**Trichome density on abaxial leaf surface**	**Trichome density on adaxial leaf surface**	**Waxy bloom**	**Plant color**
σ^2^g	(129.86)	(0.01)	28.6 (115.14)	0.14 (0.14)	0.13 (0.36)	0.05 (0.06)	(0.03)	303.35 (337.10)	535.36 (446.53)	0.19 (0.11)	0.04 (0.04)
σ^2^s	(413.37)	(0.03)	88.51	0.59 (0.39)	(0.37)	0.08 (0.05)	-	233.73 (132.26)	264.53 (216.25)	0.16	0.12 (0.13)
σ^2^r	(72.66)	-	-	0.08	(0.03)	-	-	22.55 (34.71)	42.32 (93.72)	-	-
σ^2^e	(110.81)	(0.07)	101.81 (107.64)	0.29 (0.38)	0.32 (0.12)	0.16 (0.12)	(0.06)	59.16 (104.42)	81.99 (98.31)	0.06 (0.05)	-
σ^2^a	(259.71)	(0.03)	57.21 (230.28)	0.29 (0.29)	0.26 (0.72)	0.10 (0.12)	(0.06)	606.71 (674.20)	1070.72 (893.06)	0.39 (0.22)	0.08 (0.08)
σ^2^d	(413.37)	(0.03)	88.51	0.59 (0.39)	(0.37)	0.08 (0.05)	-	233.73 (132.26)	264.53 (216.25)	0.16	0.12 (0.13)
σ^2^p	(856.54)	(0.11)	264.81 (369.66)	1.25 (1.12)	0.74 (1.24)	0.32 (0.3)	(0.12)	922.15 (945.58)	1459.56 (1301.35)	0.60 (0.28)	0.21 (0.21)
${\text{h}}_{{ns}^{2}}$	(0.30)	(0.23)	0.22 (0.62)	0.23 (0.26)	0.35 (0.58)	0.30 (0.41)	(0.51)	0.66 (0.71)	0.73 (0.69)	0.65 (0.77)	0.40 (0.39)
${\text{h}}_{b^{2}}$	(0.79)	(0.46)	0.55 (0.73)	0.70 (0.61)	0.50 (0.88)	0.55 (0.59)	(0.54)	0.91 (0.85)	0.92 (0.85)	0.91 (0.82)	0.99 (0.99)
GCA/SCA ratio	(0.31)	(0.51)	0.32	0.24 (0.37)	(0.97)	0.59 (1.13)	-	1.30 (2.55)	2.02 (2.07)	1.22	0.33 (0.32)
Predictability ratio	(0.39)	(0.51)	0.39	0.32 (0.42)	(0.66)	0.54 (0.69)	-	0.72 (0.84)	0.80 (0.81)	0.71	0.40 (0.39)

*σ^2^g, gca variance; σ^2^s, sca variance; σ^2^r, reciprocal variance; σ^2^e, environmental/error variance; σ^2^a, additive variance; σ^2^d, dominance variance; σ^2^p, phenotypic variance; ${\text{h}}_{{ns}^{2}}$, narrow sense heritability; ${\text{h}}_{b^{2}}$, broad sense heritability; GCA, general combining ability; SCA, specific combining ability; the values outside the parentheses are for rainy season, whereas inside the parentheses are for postrainy season*.

Variance due to general combining ability (σ^2^g) was higher than the variance due to specific combining ability (σ^2^s) for trichome density on abaxial and adaxial leaf surfaces across seasons; indicating the predominance of additive gene action in controlling the expression of these traits (Table 8). Trichome density on abaxial and adaxial leaf surfaces showed higher additive variance (σ^2^a) than the dominance variance (σ^2^d) across seasons. Leaf glossy score and plant vigor score in the postrainy season exhibited higher additive variance than the dominance variance. Overall resistance score exhibited high dominance variance to the additive variance across seasons. Plant vigor score and trichome density on the abaxial and adaxial leaf surfaces across seasons, and leaf glossy score in the postrainy season exhibited high GCA/SCA ratio, indicating the additive type of gene action controlling the expression of these traits. These traits also had high predictability ratios.

Heritability estimates for narrowsense (${\text{h}}_{{ns}^{2}}$) and broadsense (${\text{h}}_{b^{2}}$) heritability of all the traits ranged from 0.22 to 0.73 (Table 8). Plants with shoot fly deadhearts and overall resistance score in the rainy season, and plants with shoot fly eggs, number of shoot fly eggs/plant, and overall resistance score in the postrainy season exhibited low (≤0.30) narrowsense heritability. The other traits exhibited moderate to high narrowsense heritability. All the shoot fly resistance and morphological traits across seasons exhibited high broadsense heritability values.

## Discussion

The significant *F*-values for all the traits studied indicated the diverse nature of the genotypes used in this study. The *per se* performance of the parents and hybrids against shoot fly, *A. soccata* indicated the existence of genetic potential for shoot fly resistance in the material used. The evaluation of parents and their hybrids during the rainy and postrainy seasons indicated variation in expression of shoot fly resistance across seasons; with greater susceptibility in the rainy season (Dhillon et al., 2006c). This was largely because of available favorable environmental conditions suitable for shoot fly multiplication in the rainy season. The performance of hybrids was season specific indicating the influence of environmental factors in the expression of shoot fly resistance (Riyazaddin et al., 2015a,b). High G X E interactions for shoot fly deadhearts percentage, has been reported earlier (Padmaja et al., 2010; Aruna et al., 2011). Most of the crosses and their reciprocals showed resistance to shoot fly, suggesting that shoot fly resistance can be easily transferred into the progenies. Most of the crosses exhibited oviposition non-preference and tolerance mechanisms of resistance, which are the major components of resistance in sorghum to shoot fly (Doggett et al., 1970; Raina et al., 1981; Sharma and Nwanze, 1997; Kamatar et al., 2003; Dhillon et al., 2005, 2006b; Sivakumar et al., 2008). F_1_ hybrids based on shoot fly-resistant parents exhibited leaf glossiness, high plant vigor, leafsheath pigmentation, and high trichome density across seasons, suggesting that both the parents should be resistant to shoot fly. The genotypes ICSV 700, M 35-1, ICSV 25019, PS 35805, IS 2123, IS 2146, and IS 18551 showed resistance to shoot fly across seasons, and hence, these can be effectively utilized in developing shoot fly resistant sorghums.

Estimates of combining ability will be helpful in selecting the desirable parents for developing shoot fly-resistant hybrids. Significant mean sum of squares due to GCA for all characters, and SCA for most of the traits indicated contribution of both additive and non-additive types of gene action for resistance to shoot fly in sorghum (Borikar and Chopde, 1982; Riyazaddin et al., 2015b). Significant reciprocal mean sum of squares for plants with shoot fly eggs, overall resistance score and trichome density indicated the cytoplasmic influence in inheritance of these traits. Hence, care should be taken while selecting the parents for these traits for use in sorghum improvement. Leaf glossy score, plant vigor and trichome density had greater σ^2^g, with high GCA/SCA and predictability ratios, suggesting predominance of additive gene action in inheritance of these traits. Plants with shoot fly deadhearts, plants with shoot fly eggs, and ORS exhibited higher SCA variance, with lower GCA/SCA and predictability ratios, suggesting the predominance of non-additive (dominance) gene action in inheritance of these traits. Hence, heterosis breeding could be used for improvement of sorghum for these traits.

Predominance of different types of gene action, and their heritability differs with the shoot fly population pressure (Rana et al., 1981; Borikar and Chopde, 1982). Almost all the traits across seasons exhibited high broadsense heritabilities, indicating high inheritance of shoot fly resistance. Genotypes with significant negative *gca* effects were good combiners for shoot fly resistance. Genotypes with negative *gca* effects for plants with shoot fly eggs, number of shoot fly eggs/plant, shoot fly deadhearts, leaf glossy score, plant vigor score and leafsheath pigmentation, significant positive *gca* effects for trichome density can be selected and effectively utilized in the breeding program (Sharma et al., 1977; Hallali et al., 1982; Dhillon et al., 2006b). The crosses with negative *sca* effects for leaf glossy and plant vigor scores can be utilized in hybrid breeding process. Significant reciprocal combining ability of some of the crosses indicted influence of maternal factors in inheritance of plants with shoot fly eggs, leaf glossy score and trichome density. This information should be taken into consideration to select genotypes for use as male or female parents.

## Conclusions

The present studies indicated that the parents involved in the crossing program should possess the shoot fly resistance genes for breeding high yielding shoot fly-resistant sorghums. Both the additive and non-additive type of gene action controlled expression of resistance to shoot fly, *A. soccata*. Most of the traits exhibited high narrow and broadsense heritabilities, indicating high inheritance of the shoot fly resistance traits. Breeding for shoot fly resistance exhibited season specificity, and utmost care should be taken while selecting the parental lines. The presence of dominance gene action for shoot fly traits indicated heterosis breeding as ideal for improving shoot fly resistance in sorghum genotypes. The predominance of additive nature of gene action for leaf glossy score, plant vigor, leafsheath pigmentation and trichome density indicated simple selection techniques and recombination breeding with pedigree as methods of selection for improving these traits. The variation in expression of shoot fly resistance across seasons was due to the involvement of non-additive genetic components of resistant traits. Crosses with significant positive or negative *sca* effects for shoot fly resistance suggested that hybridization is necessary to increase the levels of shoot fly resistance. Parents involved in the crosses with significant specific combining abilities can be utilized directly in hybrid breeding process. The genotypes with good general combining ability for shoot fly resistance, and high grain yield can be used in developing shoot fly resistant cultivars for sustainable crop production.

## Author contributions

HS: Planned, supervised the research, and contributed in preparing the manuscript. RM: Executed the experiments, carried out the statistical analysis and prepared the manuscript. RM, RB: Scientific Officers, helped in carrying out the field experiments. AA: Provided financial support for field experiments and helped in preparing the manuscript. KP: Supervised the research and helped in preparing the manuscript.

### Conflict of interest statement

The authors declare that the research was conducted in the absence of any commercial or financial relationships that could be construed as a potential conflict of interest.

## References

[B1] Aruna C. Bhagwat V. R. Vittal S. Hussain T. Ghorade R. B. Khandalkar H. G. (2011). Genotype X Environment interactions for shoot fly resistance in sorghum [*Sorghum bicolor* (L.) Moench]: response of recombinant inbred lines. Crop Prot. 30, 623–630. 10.1016/j.cropro.2011.02.007

[B2] Ashok Kumar A. Reddy B. V. S. Sharma H. C. Hash C. T. Srinivasa Rao P. Ramaiah B. (2011). Recent advances in sorghum genetic enhancement research at ICRISAT. Am. J. Plant Sci. 2, 589–600. 10.4236/ajps.2011.24070

[B3] Baker R. J. (1978). Issues in diallel analysis. Crop Sci. 18, 533–536. 10.2135/cropsci1978.0011183X001800040001x

[B4] Balikai R. A. Bhagwat V. R. (2009). Evaluation of integrated pest management components for the management of shoot fly, shoot bug and aphid in rabi sorghum. Karnataka J. Agric. Sci. 22, 532–534.

[B5] Borikar S. T. Chopde P. R. (1982). Stabililty parameters for shoot fly resistance in sorghum. Indian J. Genet. 42, 155–158.

[B6] Bramel-Cox P. J. Kumar K. A. Hancock J. D. Andrews D. J. (1995). Sorghum and millets for forage and feed, in Sorghum and Millets Chemistry and Technology, ed Dendy D. A. V. (St. Paul, MN: American Association Cereal Chemists, Inc.), 325–364.

[B7] Deeming J. C. (1971). Some species of *Atherigona rondani* (Diptera: Muscidae) from northern Nigeria, with special reference to those injurious to cereal crops. Bull Entomol. Res. 61, 133–190. 10.1017/S0007485300057527

[B8] Dhillon M. K. Sharma H. C. Naresh J. S. Singh R. Pampapathy G. (2006c). Influence of cytoplasmic male sterility on expression of different mechanisms of resistance in sorghum to *Atherigona soccata* (Diptera: Muscidae). J. Econ. Entomol. 99, 1452–1461. 10.1603/0022-0493-99.4.1452 16937704

[B9] Dhillon M. K. Sharma H. C. Ram S. Naresh J. S. (2005). Mechanisms of resistance to shoot fly, *Atherigona soccata* in sorghum. Euphytica 144, 301–312. 10.1007/s10681-005-7400-4

[B10] Dhillon M. K. Sharma H. C. Reddy B. V. S. Ram S. Naresh J. S. (2006a). Inheritance of Resistance to Sorghum Shoot Fly, *Atherigona soccata*. Crop Sci. 46, 1377–1383. 10.2135/cropsci2005.06-0123

[B11] Dhillon M. K. Sharma H. C. Singh R. Naresh J. S. (2006b). Influence of cytoplasmic male-sterility on expression of physico-chemical traits associated with resistance to sorghum shoot fly, *Atherigona soccata* (Rondani). SABRAO J. Breed. Genet. 38, 105–122.

[B12] Doggett H. Starks K. J. Eberhart S. A. (1970). Breeding for resistance to the sorghum shoot fly. Crop Sci. 10, 528–531. 10.2135/cropsci1970.0011183X001000050023x

[B13] FAO (2014). Crops Primary Equivalent. Retrieved on 2nd March, 2015. Available online at: www.faostat.fao.org

[B14] GenStat (2010). Introduction to GenStat for Windows Genstat, 13th Edn. Hemel Hempstead: Lawes Agricultural Trust, Rothamsted Experimental Station.

[B15] Goyal S. N. Kumar S. (1991). Combining ability for yield component and oil content in sesame. Indian J. Genet. Plant Breed. 51, 311–314.

[B16] Griffing B. (1956). Concept of general and specific combining ability in relation to diallel crossing systems. Australian J. Biol. Sci. 9, 463–493.

[B17] Hallali M. S. Gowda B. T. S. Kulkarni K. A. Goud J. V. (1982). Inheritance of resistance to shoot fly [*Atherigona soccata* (Rond.)] in sorghum [*Sorghum bicolor* (L.) Moench]. SABRAO J. Breed. Genet. 14, 165–170.

[B18] Indostat Services (2004). Windostat. Hyderabad: Indostat Services.

[B19] Johnson H. W. Robinson H. F. Comstock R. E. (1955). Estimates of genetic and environmental variability in soybean. Agron. J. 47, 314–318. 10.2134/agronj1955.00021962004700070009x

[B20] Jotwani M. G. Teetes G. L. Young W. R. (1980). Elements of integrated control of sorghum pests, in FAO Plant Production and Protection Paper. Rome: FAO.

[B21] Kahate N. S. Raut S. M. Ulemale P. H. Bhogave A. F. (2014). Management of Sorghum Shoot Fly. Popular Kheti. 2, 72–74.

[B22] Kamatar M. Y. Salimath P. M. Kumar R. L. R. Rao T. S. (2003). Heterosis for biochemical traits governing resistance to shoot fly in sorghum [*Sorghum bicolor* (L.) Moench]. Indian J. Genet. 63, 124–127.

[B23] Kumar A. A. Reddy B. V. S. Sharma H. C. Ramaiah B. (2008). Shoot fly (*Atherigona soccata*) resistance in improved grain sorghum hybrids. *E J. SAT Agric*. Res. 6, 1–4.

[B24] Maiti R. K. Bidinger F. R. (1979). Simple approaches to the identification of shoot fly tolerance in sorghum. Indian J. Plant Prot. 7, 135–140.

[B25] Mohammed R. Are A. K. Bhavanasi R. Munghate R. S. Kavi Kishor P. B. Sharma H. C. (2015). Quantitative genetic analysis of agronomic and morphological traits in sorghum, *Sorghum bicolor*. Front. Plant Sci. 6:945. 10.3389/fpls.2015.00945 26579183 PMC4630571

[B26] Padmaja P. G. Madhusudhana R. Seetharama N. (2010). Sorghum Shoot Fly. Hyderabad: Directorate of Sorghum Research.

[B27] Prabhakar and Raut M. S. (2010). Exploitation of heterosis using diverse parental lines in Rabi Sorghum. Elec. J. Plant Breed. 1, 680–684.

[B28] Raina A. K. Thindwa H. Z. Othieno S. M. Corkhill R. T. (1981). Resistance in sorghum to sorghum shoot fly: larval development and adult longevity and fecundity on selected cultivars. Insect Sci. Appl. 2, 99–103.

[B29] Rana B. S. Jotwani M. G. Rao N. G. P. (1981). Inheritance of host plant resistance to sorghum shoot fly. Insect Sci. Appl. 2, 105–100.

[B30] Riyazaddin M. D. Kavi Kishor P. B Are A. K. Reddy B. V. S. Rajendra S. M. Sharma H. C. (2015a). Mechanisms and diversity of resistance to sorghum shoot fly, *Atherigona soccata*. Plant Breed. 134, 423–436. 10.1111/pbr.12276

[B31] Riyazaddin M. D. Munghate R. S. Are A. K. Kavi Kishor P. B Reddy B. V. S. Sharma H. C. (2015b). Components of resistance to sorghum shoot fly, *Atherigona soccata*. Euphytica 207, 419–438. 10.1007/s10681-015-1566-1

[B32] Sharma G. C. Jotwani M. G. Rana B. S. Rao N. G. P. (1977). Resistance to the sorghum shoot fly, *Atherigona soccata* (Rondani) and its genetic analysis. J. Entomol. Res. 1, 1–12.

[B33] Sharma H. C. Nwanze K. F. (1997). Mechanisms of Resistance to Insects and their Usefulness in Sorghum Improvement. Information Bulletin no. 55, Patancheru: International Crops Research Institute for the Semi-Arid Tropics (ICRISAT).

[B34] Sharma H. C. Taneja S. L. Kameswara Rao N. Prasada Rao K. E. (2003). Evaluation of Sorghum Germplasm for Resistance to Insect Pests. Information Bulletin no. 63, Patancheru: International Crops Research Institute for the Semi-Arid Tropics (ICRISAT).

[B35] Sharma H. C. Taneja S. L. Leuschner K. Nwanze K. F. (1992). Techniques to Screen Sorghums for Resistance to Insect Pests. Information Bulletin no. 32. Patancheru: International crops research institute for the Semi-Arid tropics (ICRISAT).

[B36] Sharma H. C. (1985). Future strategies for pest control in sorghum in India. Trop. Pest Manag. 31, 167–185. 10.1080/09670878509370977

[B37] Sharma H. C. (1993). Host-Plant Resistance to insects in sorghum and its role in integrated pest management. Crop Prot. 12, 11–34. 10.1016/0261-2194(93)90015-B

[B38] Singh R. K. Chaudhary B. D. (1985). Biometrical methods in quantitative genetics. New Dehli: Kalyani Publishers, 102–127.

[B39] Sivakumar C. Sharma H. C. Lakshmi Narasu M. Pampapathy G. (2008). Mechanisms and diversity of resistance to shoot fly, *Atherigona soccata* in *Sorghum bicolor*. Indian J. Plant Prot. 36, 249–256.

[B40] Soto P. E. (1974). Ovipositional preference and antibiosis in relation to resistance to sorghum shoot fly. J. Econ. Entomol. 67, 265–267. 10.1093/jee/67.2.265 4406654

